# NTRK*-*fused central nervous system tumours: clinicopathological and genetic insights and response to TRK inhibitors

**DOI:** 10.1186/s40478-024-01798-9

**Published:** 2024-07-16

**Authors:** Eric Eunshik Kim, Chul-Kee Park, Seung-Ki Kim, Ji Hoon Phi, Sun Ha Paek, Jung Yoon Choi, Hyoung Jin Kang, Joo Ho Lee, Jae Kyung Won, Hongseok Yun, Sung-Hye Park

**Affiliations:** 1 Department of Pathology, Seoul National University College of Medicine, 101 Daehak-Ro, Jongno-Gu, Seoul, Republic of Korea; 2 Department of Neurosurgery, Seoul National University Hospital, Seoul, Republic of Korea; 3 Department of Pediatrics, Seoul National University Hospital, Seoul, Republic of Korea; 4 Department of Radiation Oncology, Seoul National University Hospital, Seoul, Republic of Korea; 5 Department of Genomic Medicine, Seoul National University Hospital, Seoul, Republic of Korea; 6 Neuroscience Research Institute, Seoul National University Hospital, Seoul, Republic of Korea; 7 Seoul National University Cancer Research Institute, Seoul, Republic of Korea

**Keywords:** Brain tumours, Next-generation sequencing, *NTRK* fusion, TRK inhibitors

## Abstract

*Background* Neurotrophic tropomyosin receptor kinase (*NTRK*) gene fusions are found in 1% of gliomas across children and adults. TRK inhibitors are promising therapeutic agents for NTRK-fused gliomas because they are tissue agnostic and cross the blood–brain barrier (BBB). *Methods* We investigated twelve NGS-verified NTRK-fused gliomas from a single institute, Seoul National University Hospital. *Results* The patient cohort included six children (aged 1–15 years) and six adults (aged 27–72 years). *NTRK2* fusions were found in ten cerebral diffuse low-grade and high-grade gliomas (DLGGs and DHGGs, respectively), and *NTRK1* fusions were found in one cerebral desmoplastic infantile ganglioglioma and one spinal DHGG. In this series, the fusion partners of *NTRK2* were *HOOK3*, *KIF5A, GKAP1, LHFPL3, SLMAP, ZBTB43*, *SPECC1L, FKBP15, KANK1,* and *BCR,* while the *NTRK1* fusion partners were *TPR and TPM3.* DLGGs tended to harbour only an *NTRK* fusion, while DHGGs exhibited further genetic alterations, such as *TERT* promoter/*TP53*/*PTEN* mutation, *CDKN2A/2B* homozygous deletion*, PDGFRA/KIT/MDM4/AKT3* amplification, or multiple chromosomal copy number aberrations. Four patients received adjuvant TRK inhibitor therapy (larotrectinib, repotrectinib, or entrectinib), among which three also received chemotherapy (n = 2) or proton therapy (n = 1). The treatment outcomes for patients receiving TRK inhibitors varied: one child who received larotrectinib for residual DLGG maintained stable disease. In contrast, another child with DHGG in the spinal cord experienced multiple instances of tumour recurrence. Despite treatment with larotrectinib, ultimately, the child died as a result of tumour progression. An adult patient with glioblastoma (GBM) treated with entrectinib also experienced tumour progression and eventually died. However, there was a successful outcome for a paediatric patient with DHGG who, after a second gross total tumour removal followed by repotrectinib treatment, showed no evidence of disease. This patient had previously experienced relapse after the initial surgery and underwent autologous peripheral blood stem cell therapy with carboplatin/thiotepa and proton therapy. *Conclusions* Our study clarifies the distinct differences in the pathology and TRK inhibitor response between LGG and HGG with *NTRK* fusions.

**Supplementary Information:**

The online version contains supplementary material available at 10.1186/s40478-024-01798-9.

## Introduction

Neurotrophic tropomyosin receptor kinase (*NTRK*) family gene fusions are rare, found in only 1% of gliomas across adult and paediatric patients. Despite the rarity of these fusions, TRK inhibitors are emerging as promising therapeutic options. This promise stems from their broad efficacy across various cancer types of any organ, their ability to cross the blood‒brain barrier (BBB) effectively, and the ongoing improvement of second-generation inhibitors designed to overcome resistance [7, 16, 22, 32].

TRK inhibitors represent a targeted strategy for cancer therapy designed to block the activity of *NTRK* fusion proteins. These fusion proteins are formed when the *NTRK* family genes *NTRK1*, *NTRK2*, and *NTRK3* fuse with other genes, leading to abnormal activation of signalling pathways that promote cancer cell growth and survival [10]. TRK inhibitors achieve these effects by binding to the ATP binding site of *NTRK* fusion proteins, preventing the phosphorylation of downstream signalling molecules and blocking the transmission of growth signals within cancer cells (Table 1) [16]. The mechanistic basis of TRK inhibitors lies in their disruption of signalling pathways responsible for driving cancer cell proliferation and survival, ultimately leading to tumour regression or tumour growth inhibition [33].

**Table 1:** Summary of the mechanism of action, targets, pharmaceutical effects, and clinical trial results of first-generation and second-generation NTRK inhibitors, including larotrectinib, repotrectinib, or entrectinib

Aspect	Larotrectinib	Entrectinib	Repotrectinib
FDA approval year	November 26, 2018	August 15, 2019	November 15, 2023
Mechanism of action	Inhibits TRK fusion proteins, disrupting downstream signaling pathways	Blocks TRK fusion proteins,disrupting aberrant signaling cascade	Targets TRK fusion proteins, ROS1, and ALK, inhibiting signaling pathways
Targets	TRKA/B/C fusion proteins	TRK A/B/C fusion proteins, ROS1, ALK	TRKA/B/C fusion proteins, ROS1, ALK
Binding site	ATP-binding site (Type 1 kinase inhibitor)	ATP-binding site (Type 1 kinase inhibitor)	Allosteric binding site (Type 3 kinase inhibitor)
Pharmaceutical effects	Induces tumour regression in NTRK fusion-positive cancers	Exhibits efficacy in NTRK fusion-positive cancers	Active against NTRK, ROS1, and ALK-positive cancers
Clinical trial results	Demonstrates high response rates in clinical trials	Showed effectiveness in various cancer types	Demonstrates promising activity in clinical trials

In 2018, larotrectinib became the initial TRK inhibitor to gain FDA approval, demonstrating selective inhibition of TRK A/B/C across various solid tumours, including gliomas [8]. Notably, larotrectinib exhibited good tolerance in paediatric patients, with minimal adverse effects such as mild vomiting and fever. Subsequently, in 2019, entrectinib received FDA approval for TRK A/B/C inhibition in NTRK-fused solid tumours and ROS1-positive nonsmall cell lung cnancer (NSCLC) [5]. However, resistance to larotrectinib and entrectinib has emerged, as has been the case for other tyrosine kinase inhibitors, leading to FDA approval of the first second-generation TRK inhibitor, repotrectinib, in 2023 [9], however, in other solid cancer types, it is currently undergoing clinical trials (TPX-0005).

Despite the increasing interest in TRK inhibitors, the diagnosis of tumours with *NTRK* fusions presents numerous challenges. Due to the rarity of *NTRK* fusions in all cancers, routine next-generation sequencing (NGS) is impractical for all solid tumours [10]. The use of alternative molecular testing methods such as direct sequencing or in situ hybridization is not recommended because of the diversity of *NTRK* fusions observed in various central nervous system (CNS) tumours, with *NTRK1*/2/3 interacting with multiple fusion partners [10]. Pan-TRK immunohistochemistry (IHC) using antibodies such as EPR17341 (Ventana, ready to use, Export, US) and A7H6R (Cell Signaling, 1:50, Massachusetts, US) is available but has exhibited inconsistent reliability as a screening tool for *NTRK* fusions [2, 25]. Until pan-TRK IHC is adequately optimized to serve as a surrogate marker for *NTRK* fusion, NGS remains the most precise method for confirming the diagnosis of NTRK-fused tumours. This approach is the foundation for the evidence-based administration of TRK inhibitors.

There is a compelling rationale to continue pursuing efficient and effective diagnostic methods for NTRK-fused tumours, particularly for CNS tumours. The BBB has historically been an obstacle to successful pharmacotherapy for CNS tumours. Therefore, it is essential to explore molecules with the ability to effectively penetrate the BBB, maximally extending their utility. The ability of TRK inhibitors to cross the BBB provides a primary incentive to optimize the detection of NTRK-fused CNS tumours [5, 8]. Second, both neuropathologists and oncologists emphasize the effectiveness of TRK inhibitors against solid tumours that have metastasized to the brain [8].

For patients with high-grade gliomas (HGGs), the standard treatment approach, which includes a combination of surgery, concurrent chemoradiation therapy and Temozolomide (CCRT-TMZ), still results in a poor prognosis. This consideration has prompted the exploration of alternative therapeutic options [30]. For paediatric glioma patients, the urgent need for a targeted therapy is increased because radiation therapy carries the inherent risks of the development of secondary glioma, meningioma, or sarcoma and radiotoxicity to the developing and growing brain [3, 13]. This study was designed to investigate the real-world applications of the diagnostic and therapeutic options for NTRK-fused gliomas in both paediatric and adult patients.

## Materials and methods

Between 2018 and January 2024, twelve patients with confirmed NTRK*-*fused gliomas were identified at Seoul National University Hospital (SNUH). Since the establishment of the NGS platform and brain tumour-targeted gene panel in the Department of Pathology at SNUH in 2018, nearly all primary CNS tumours have been subjected to NGS. Since the initiation of this NGS program, twelve cases have been discovered, including two recurrent cases. The initial tumours in two specific patients developed in 1998 and 2017. In this study, the clinicopathological characteristics of the twelve patients with NTRK-fused CNS tumours were reviewed, highlighting the sex and age distributions of the patients (Tables 2 and 3). The paediatric patients showed a significant female predominance, with a male:female ratio of 1:5 and a median age of 3 years (range, 1–15 years). In contrast, the adult patients exhibited a male predominance, with a male:female ratio of 4:2 and a median age of 64 years (range, 27–72 years). Overall, the female:male ratio was 7:5. In terms of tumour grade, LGGs were more common among female patients (female:male ratio of 5:1) and paediatric patients (child:adult ratio of 4:2), while HGGs were more prevalent among male patients (male:female ratio of 4:2) and adult patients (child:adult ratio of 2:4).

**Table 2:** Clinicopathologic characteristics

Patient characteristics	Children	Adults
Total No. (N = 11)	6	6
Sex		
Male	1	4
Female	5	2
Ag, median, years		
≤ 3	4	0
Teenage	2	0
Young adult < 35	0	2
Adult ≥ 35	0	4
Location		
Hemisphere	3	4
Lateral ventricle or thalamus	2	0
Cerebellum	0	2
Spinal cord	1	0
Fusion genes		
*NTRK2*	*4*	*6*
*NTRK1*	*2*	*0*
Treatment		
Operation only	2	4
OP+CCRT	1	0
OP+CCRT+TMZ	0	1
OP+ NTRK inhibitor	1	0
OP+Adjuvant Tx+NTRK inhibitor	2	1

**Table 3:** Clinical features of eleven SNUH patients with *NTRK*-fused gliomas

No	Sex	Age (Year)	Site	Grade	Histopathological classification	Gene fusion	Additional genetic alterations	Post-OP Tx	F-U	Outcomes
1	F	14	Rt P	LG	DLGG	*HOOK3::NTRK2*	Absent	None	2 yrs	NET
2	F	15	Rt LV & 3rd V	LG	MGNT-like	*KIF5A::NTRK2*	Absent	None	1 mo	Stable
3	F	3	Rt Thal, 3rd V	LG	DLGG	*GKAP1::NTRK2*	Absent	Larotrectinib	10 mos	Stable
4	M	1	Lt TO	LG	DIG	*TPR::NTRK1*	Absent	OP (× 2), CCRT	25 yrs	NET
5	F	27	Lt T	LG	Astrocytoma with Neuropil-like islands-like	*LHFPL2::NTRK2*	Absent	None	13 yrs	NET
6	F	31	Rt F	LG	DLGG	*SLMAP-NTRK2*	Absent except BRCA1 (Q206*)	None	5 mos	NET
7	F	2	Lt FT	HG	IHG	*ZBTB43::NTRK2*	CDKN2A/2B HemiD	Proton Tx, 2nd OP, Repotrectinib	3 yrs	NET
8	F	2	C7-L1	HG	GBM-like	*TPM3::NTRK1*	CDKN2A/2B HoD MDM4/AKT3 amp, PTEN/FGFR2 loss	CCRT-TMZ #2, 2nd OP, PCV #4, 3rd OP, 4th OP, Larotrectinib	4.3 yrs	Progressive, Death (OS post–larotrectinib: 22 mos)
9	M	64	Cerebellar tonsil	HG	GBM-like	*SPECC1L::NTRK2*	CDKN2A HoD CDKN2B HemiD	GTR-CCRT-TMZ #6	27 mos	Stable
10	M	67	Lt cerebellum	HG	GBM-like	*FKBP15::NTRK2*	CDKN2A/2B HoD, PDGFRA/KIT amp, PTEN/NF1 loss, SMARCA2 HoD	CCRT-TMZ #6, Entrectinib	21.5 mos	Progression, Death
11	M	72	Rt T	HG	GBM-like	*KANK1::NTRK2*	CDKN2A/2B HoD TERTp (C228T), PTEN & TP53 mut PTEN loss, 7 trisomy, loss of 10, 14q, 22q	None, due to poor general condition	4 mos	Death (OS: 4 mos)
12	M	54	Lt Thal, Bilat. F	HG	GBM-like	*BCR::NTRK2*	CDKN2A/2B HoD TERTp (C228T), PTEN mut	None refused treatment	13 mos	Death (OS: 13 mos)

*P* Parietal; *LV* Lateral ventricle; *3rd V* Third ventricle; *Thal* Thalamus; *T* Temporal; *PO* Parieto-occipital lobe; *FT* Frontotemporal; *Bilat. F* Bilateral frontal; *IHG*, Infant-type hemispheric glioma; *LG* Low-grade; *HG* High-grade; *DLGG* Diffuse low-grade glioma; *MGNT* Myxoid glioneuronal tumour; *DIG* Desmoplastic infantile ganglioglioma; *IHG* Infant-type hemispheric glioma; *DHGG* Diffuse high-grade glioma; *GBM IDH-wt* Glioblastoma, IDH-wildtype; *HemiD* Hemizygous deletion, *HoD* Homozygous deletion; loss, 1 copy loss; *Mut* Mutation; *OP* Operation; *CCRT-TMZ* Concurrent chemoradiotherapy with temozolomide; *PCV* Procarbazine + lomustine + vincristine chemotherapy; *mos* Months; *NET* No evidence of tumour; *OS* Overall survival

### Immunohistochemical analysis

Neutral formalin-fixed, paraffin-embedded (FFPE) tissues were sliced into 3 μm thick sections for H&E staining and IHC. For pan-TRK IHC, two different clones were used for each tumour. The pan-TRK assays with EPR17341 (Ventana, ready to use, Export, US) and A7H6R (Cell Signaling, 1:50, Massachusetts, US) were performed using a standard avidin–biotin-peroxidase method with the BenchMark ULTRA procedure (Roche Diagnostics). For the pan-TRK assay with the Cell Signaling antibody (clone A7H6R), the vendor-recommended immunoprecipitation antibody dilution of 1:50 was used, with antigen retrieval for 64 min and antibody incubation for 60 min. Tumours were classified as positive for TRK if more than 1% of the tumour cells exhibited staining at any intensity above the background intensity, regardless of the subcellular staining pattern (cytoplasmic, membranous, nuclear, or perinuclear). However, in TRK-positive tumours, the staining was consistently diffuse and robustly positive, observed in more than 90% of the cells [11]. For diagnosis, several immunohistochemical analyses were conducted on tumour sections using the following antibodies (Supplementary Table 1): anti-IDH1 R132H (H09) monoclonal antibody (1:100 dilution, Dianova, Hamburg, Germany), anti-ATRX polyclonal antibody HPA001906 (1:300 dilution, Atlas Antibodies AB, Bromma, Sweden), anti-p53 monoclonal antibody, DO-7 code M7001 (1:1000 dilution, DAKO, Glostrup, Denmark), anti-pHH3 antibody (1:100 dilution, Cell Marque, Rocklin, USA), and anti-Ki67 antibody (1:1000 dilution, DAKO, Glostrup, Denmark). The Ki-67 labelling index was calculated using the Sectra Ki-67 morphometric analyser on virtual Leica Biosystems slides. We used known positive tissues or internal positive controls as IHC-positive controls; for the negative controls, the primary antibodies were omitted during IHC.

### NGS and pipelines for the analysis of somatic mutations

NGS analyses were performed with tumour DNA extracted from FFPE tumour tissues and the NEXTSeq 550Dx system using a tailored panel called “FiRST Brain Tumour DNA/RNA panel v3.3”, which was developed in-house at SNUH, as previously reported by our research team [20]. The panel comprises 223 brain tumour-associated genes and 151 fusion genes, including *NTRK1/2/3*. The fusion genes were sequenced using RNA methods. Somatic mutations were detected using the Genome Analysis Toolkit (GATK) Mutect2 v4.1.4.1. tool with the default parameters. To avoid germline variant contamination, we used the gnomad.hg19.vcf Genome Aggregation Database (gnomAD) and 1000 g_pon.hg19.vcf files, which included a normal panel for 1000 genomes. The files were provided by the GATK resource bundle. After somatic mutations were called, all variants were annotated with ANNOVAR (https://doc-openbio.readthedocs.io/projects/annovar/en/latest/) as previously described [20].

## Results

### Histopathology of NTRK-fused gliomas

NTRK-fused gliomas can be categorized as DLGGs or DHGGs based on the mitotic rate, microvascular proliferation (MVP) status, and necrosis status (Supplementary Table 2). The Ki-67 labelling index was used as a reference.

### Diffuse low-grade gliomas (DLGGs)

In this cohort, the majority of DLGGs with NTRK fusions exhibited diffusely infiltrating astrocytic tumours characterized by low mitotic activity (< 3/10 high-power fields (HPFs)) and a low Ki-67 labelling index (< 3%). These tumours lacked HG features, such as MVP and necrosis. However, three cases displayed distinctive features that were morphologically similar to those of myxoid glioneuronal tumour (MGNT) of the lateral ventricle, desmoplastic infantile ganglioglioma (DIG), and astrocytoma with neuropil-like islands, as described in the 2016 4th edition of the WHO Classification of Tumours of the Central Nervous System (hereafter referred to as the WHO2016).

**Fig. 1:**
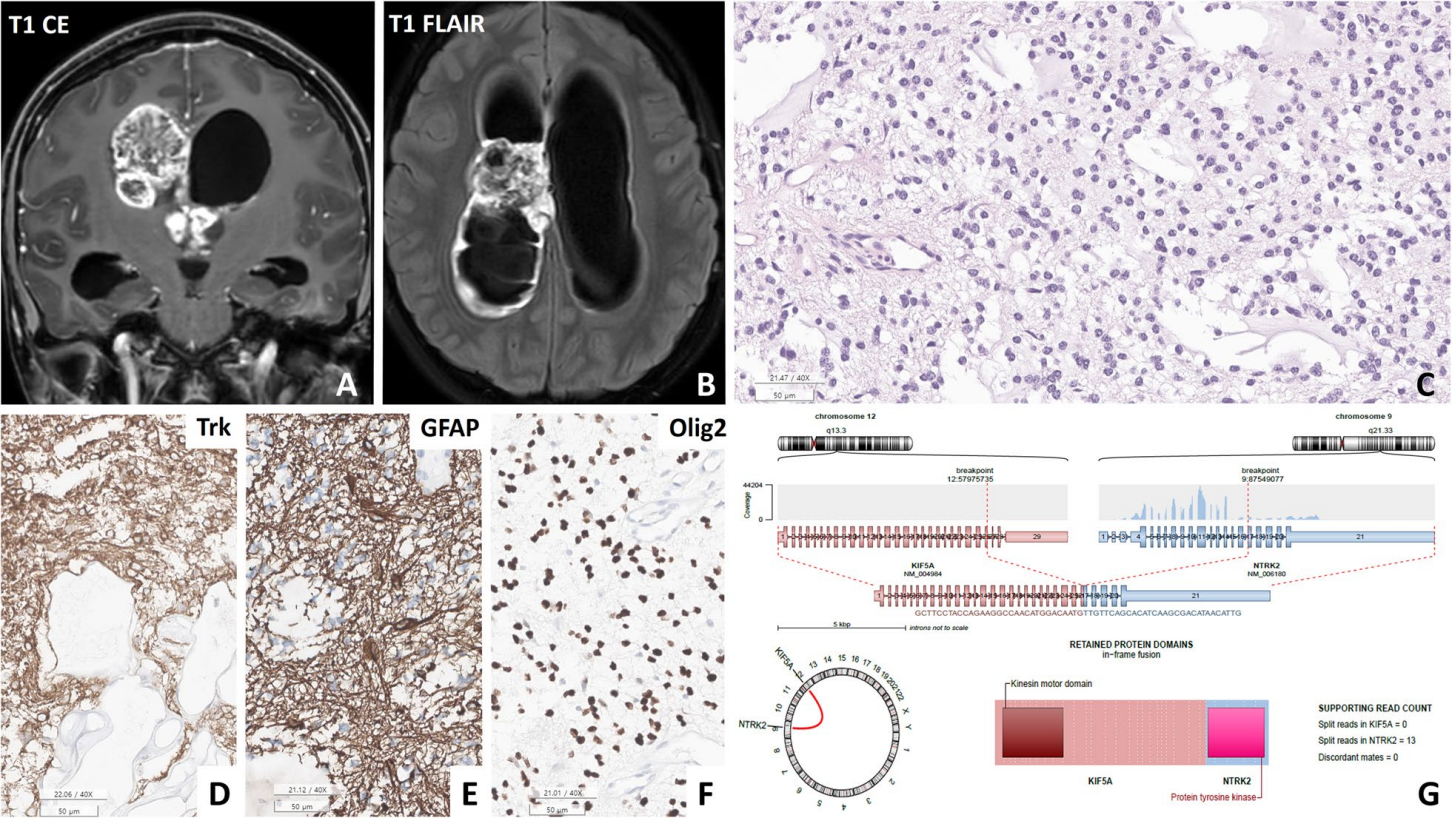
**A**, **B** Patient #2 (15 y/F) presented with an intraventricular diffuse low-grade glioma located at the lateral ventricle, exhibiting heterogeneous high signal intensity on contrast-enhanced T1-weighted and T1-fluid-attenuated inversion recovery (FLAIR) images. **B** The tissue in the H&E section resembled a myxoid glioneuronal tumour, characterized by monotonous round cells within a myxoid background. **D**, **F** Immunohistochemical analysis revealed positivity for GFAP, TRK, and Olig2 in this tumour. **G** An Arriba plot generated from next-generation sequencing (NGS) data using RNA identified the *KIF5A::NTRK2* in-frame fusion in the tumour (**C**, **D**: H&E, **E**: GFAP, **F**: Olig2; scale bar, **C**-**E**: 200 μm, **F**: 100 μm)

A tumour with the *KIF5A::NTRK2* fusion that developed in the right lateral and 3rd ventricles of patient #2 (15 y/F) exhibited features of a LG MGNT (Fig. 1).

A tumour with the *TPR::NTRK1* fusion that developed in the left temporo-occipital lobe of patient #4 (1 y/F) exhibited microscopic features compatible with DIG, CNS WHO grade 1. This patient is a long-term survivor with no evidence of tumour (NET) for 25 years following gross total resection. The tumour exhibited storiform spindled astrocytic tumour cells and ganglion cells, fitting the profile for DIG.

**Fig. 2:**
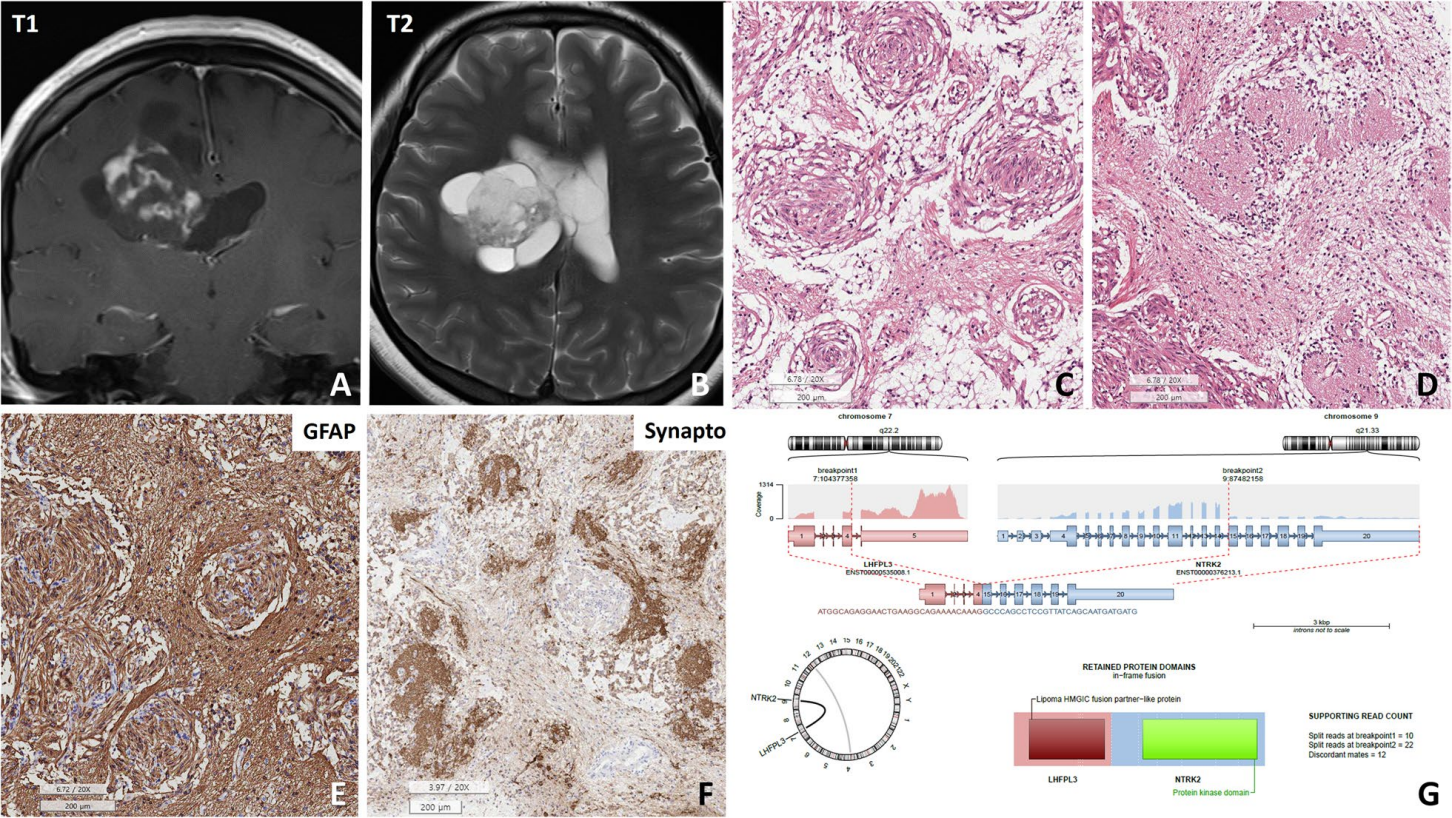
Patient #5, a 27-year-old female, presented with an uncommon glioma harbouring the *LHFPL3::NTRK2* fusion*.* **A**, **B** T1 and T2 FLAIR MR images revealed a 5.3 × 4.5 × 1.6 cm solid and cystic mass in the right lateral ventricle with multifocal enhancement in the right intra- and periventricular white matter. **C**, **D** H&E sections of the tumour exhibited a distinctive pathology characterized by multiple whorls formed by glial cells and neuropil-like islands. **E** The spindle-shaped glial cells forming the whorls were positive for GFAP and negative for synaptophysin, but **F** the neuropil-like islands were positive for synaptophysin and negative for GFAP. **G** An Arriba plot generated from next-generation sequencing (NGS) data for tumour RNA identified the *LHFPL3::NTRK2* in-frame fusion (scale bar, **C**–**E**: 200 μm, **F**: 100 μm)

A tumour with the *LHFPL2::NTRK2* fusion in the left temporal lobe of patient #5 (27 y/F) had features of anaplastic astrocytoma with neuropil-like islands, on the basis of the diagnostic criteria of the WHO2016 classification. The neuropil-like islands were positive for synaptophysin but negative for GFAP (Fig. 2). This tumour had a moderately high mitotic rate (4/10 HPFs) but a low Ki-67 index (1.2%).

### Diffuse high-grade gliomas (DHGGs)

The histopathological characteristics of four adult HGGs (patients #9-#12) with *SPECC1L::NTRK2, FKBP15::NTRK2, KANK1::NTRK2,* and *BCR::NTRK2* fusions and a paediatric HGG with the *ZBTB43::NTRK2* fusion (patient #7, 2 y/F) were consistent with IDH wild-type (IDH-wt) GBM. These tumours displayed high mitotic activity, high Ki-67 proliferation indices, MVP, and necrosis, meeting the criteria for CNS WHO grade 4 tumours.

A spinal cord tumour with the *TPM3::NTRK1* fusion (patient #8, 2y/F) also exhibited a GBM-like pathology with MVP and necrosis.

**Fig. 3:**
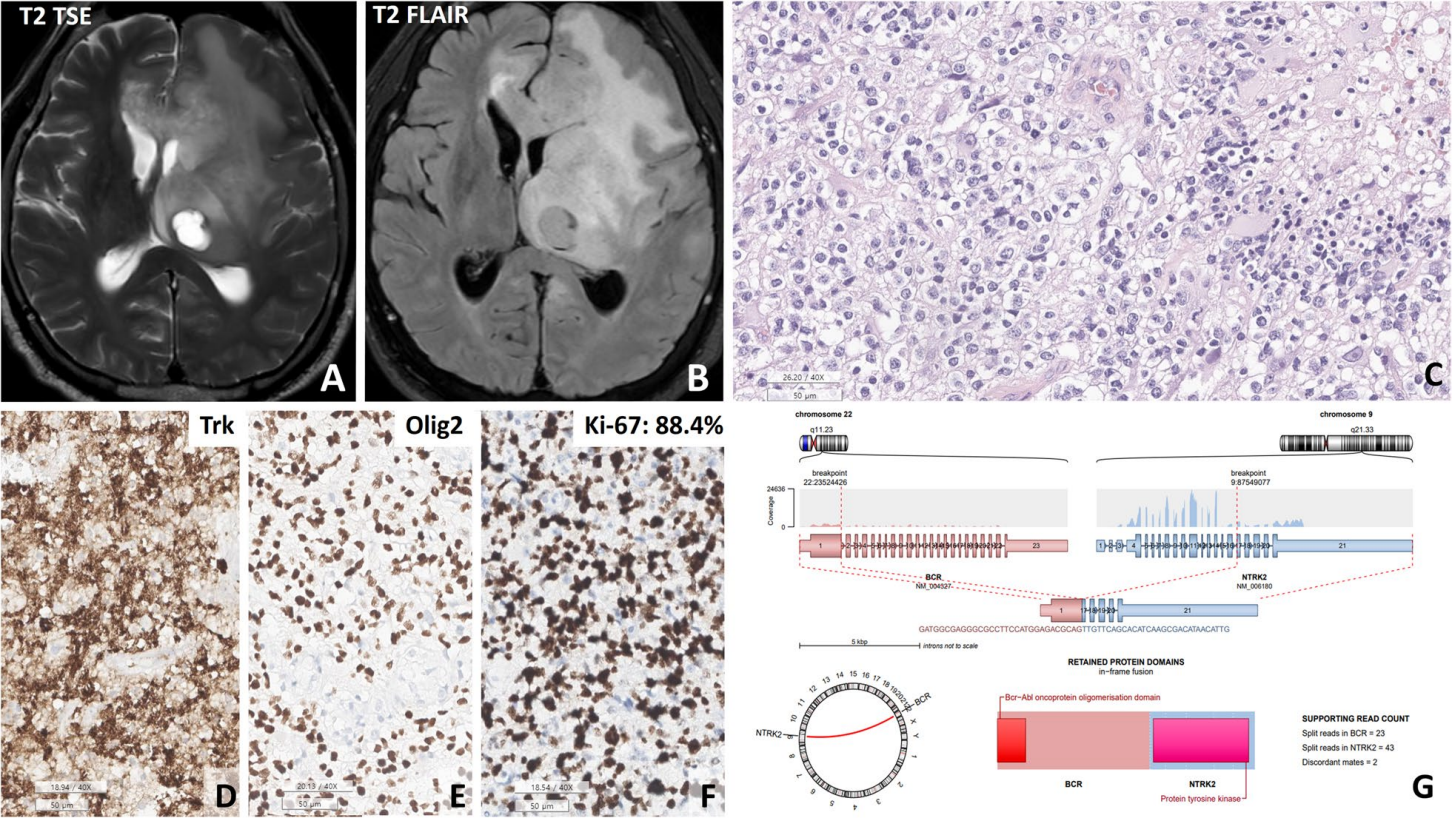
Patient #12 had the *BCR::NTRK2* fusion. **A**, **B** T2-weighted turbo spin‒echo (TSE) and T2 FLAIR images showing a hyperintense mass in the thalamus and bifrontal area with peritumoral oedema. **C** The tumour was composed of oligodendroglioma-like clear cells with microvascular proliferation and pseudopalisading necrosis. **D** and **E** Diffuse positivity for TRK and Olig2 was observed in the cytoplasm and nuclei, respectively, of tumour cells. **F** The Ki-67 labelling index was high (88.4%). **G** The Arriba plot revealed the in-frame fusion between the *BCR* gene and *NTRK2* gene (scale bar, **F**: 50 μm)

One GBM, IDH-wt with the *BCR::NTRK2* fusion, in patient #12 (54 y/M) contained uniform round cells with a clear cytoplasm, resembling those of oligodendroglioma. This tumour also harboured additional genetic abnormalities, including TERT promoter (C228T) mutation, PTEN mutation, and homozygous CDKN2A/2B deletion (Fig. 3).

This categorization scheme emphasizes the differences in histological features and molecular profiles between LGG and HGG in patients with NTRK-fused gliomas.

In the NTRK*-*fused CNS tumours in our patients, pan-TRK IHC using clone A7H6R and clone EPR17341 revealed diffuse cytoplasmic positivity with sparing of nuclei in nearly 100% of the tumour cells across all cases. Clone EPR17341 displayed slightly less intense staining than clone A7H6R, but both clones showed good concordance with the *NTRK* fusion.

### Genetic abnormalities of NTRK-fused gliomas

Except for the DIG of patient #4 (1 y/F) and the spinal HGG of patient #6 (2 y/F), both of which had *NTRK1* fusions, all other cerebral gliomas had *NTRK2* fusions. The fusion partners of *NTRK2* included *HOOK3*, *KIF5A, GKAP1, LHFPL3, SLMAP, ZBTB43*, *SPECC1L, FKBP15, KANK1,* and *BCR,* while the *NTRK1* fusion partners were *TPR* and *TPM3* (Table 4).

**Table 4:** Information on the NTRK fusion genes observed in this study

No	Sex	Age (Year)	Grade	Gene fusion	Braking point cytobands	Transcript1	Transcript2	Last	First
1	F	14	1	*HOOK3::NTRK2*	8p11.21 | 9q21.33	NM_032410	NM_001018064	EXON_13	EXON_13
2	F	15	1	*KIF5A::NTRK2*	12q13.3 | 9q21.33	NM_004984	NM_006180	EXON_ 26	EXON_17
3	F	3	1	*GKAP1::NTRK2*	9q21.32 | 9q21.33	NM_025211	NM_006180	EXON_10	EXON_16
4	F	1	1	*TPR::NTRK1*	1q31.1 I 1q23.1	NM_003292	NM_002529	EXON_24	EXON_11
5	F	27	1	*LHFPL2::NTRK2*	7q22.2 I 9q21.33	NM_199000	NM_006180	EXON_4	EXON_15
6	F	31	1	*SLMAP-NTRK2*	3p14.3 | 9q21.33	NM_007159	NM_006180	EXON_11	EXON_16
7	F	2	4	*ZBTB43::NTRK2*	9q33.3 | 9q21.33	NM_014007	NM_006180	EXON_3	EXON_15
8	F	2	4	*TPM3::NTRK1*	1q21.3 | 1q23.1	NM_152263	NM_002529	EXON_8	EXON_10
9	M	64	4	*SPECC1L::NTRK2*	22q11.23 | 9q21.33	NM_015330	NM_006180	EXON_11	EXON_17
10	M	67	4	*FKBP15::NTRK2*	9q32 | 9q21.33	NM_015258	NM_006180	EXON_20	EXON_15
11	M	72	4	*KANK1::NTRK2*	9p24.3 | 9q21.33	NM_015158	NM_001018064	EXON_3	EXON_12
12	M	54	4	*BCR::NTRK2*	22q11.23 | 9q21.33	NM_004327	NM_006180	EXON_1	EXON_17

### Diffuse low-grade gliomas (DLGGs)

All LGGs exclusively harboured *NTRK* fusions without additional genetic alterations.

### Diffuse high-grade gliomas (DHGGs)

The HGGs, including the GBMs in the adult patients and a spinal HGG in a paediatric patient, exhibited additional genetic alterations. The genetics of IHG in patient #7 was relatively simple and had hemizygous deletion of CDKN2A/2B in addition to ZBTB43::NTRK2 fusion.

**Fig. 4:**
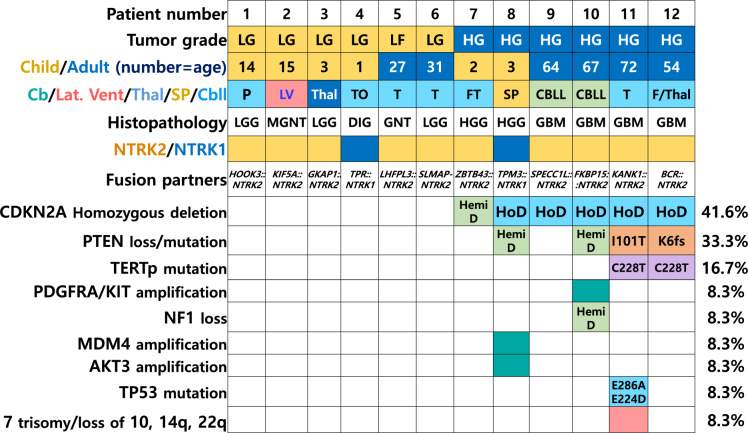
Illustration of the clinicopathological and genetic abnormalities in *NTRK*-fused gliomas in paediatric and adult patients, Abbreviations LG, low-grade; HG, high-grade; P, parietal lobe; LV, lateral ventricle; Thal, thalamus, TO, temporo-occipital lobe; T, temporal lobe; FT, fronto-temporal lobe; SP, spinal cord; CBLL, cerebellum; F/Thal, frontal lobe and thalamus; Hemi D, hemizygous deletion; HoD, homozygous deletion

In the remaining tumours, additional genetic alterations included homozygous deletion of *CDKN2A*/*2B* in five patients (#8-12), *TERT* promoter (C228T) mutation in two patients (#11 and #12), *PDGFRA/KIT/MDM4/AKT3* amplification in patient #10, and mutations in *TP53 (p.Glu286Ala, c.857A* > *C*; *p.Glu224Asp, c.672G* > *T*) and *PTEN* (p.Ile101Thr, c.302 T > C; p.Lys6fs, c.17_18delAA) as well as multiple chromosomal losses and trisomy 7 in patient #11 (Table 3 and Fig. 4).

### Treatment and response to TRK inhibitor therapy in patients with NTRK-fused gliomas

**Fig. 5:**
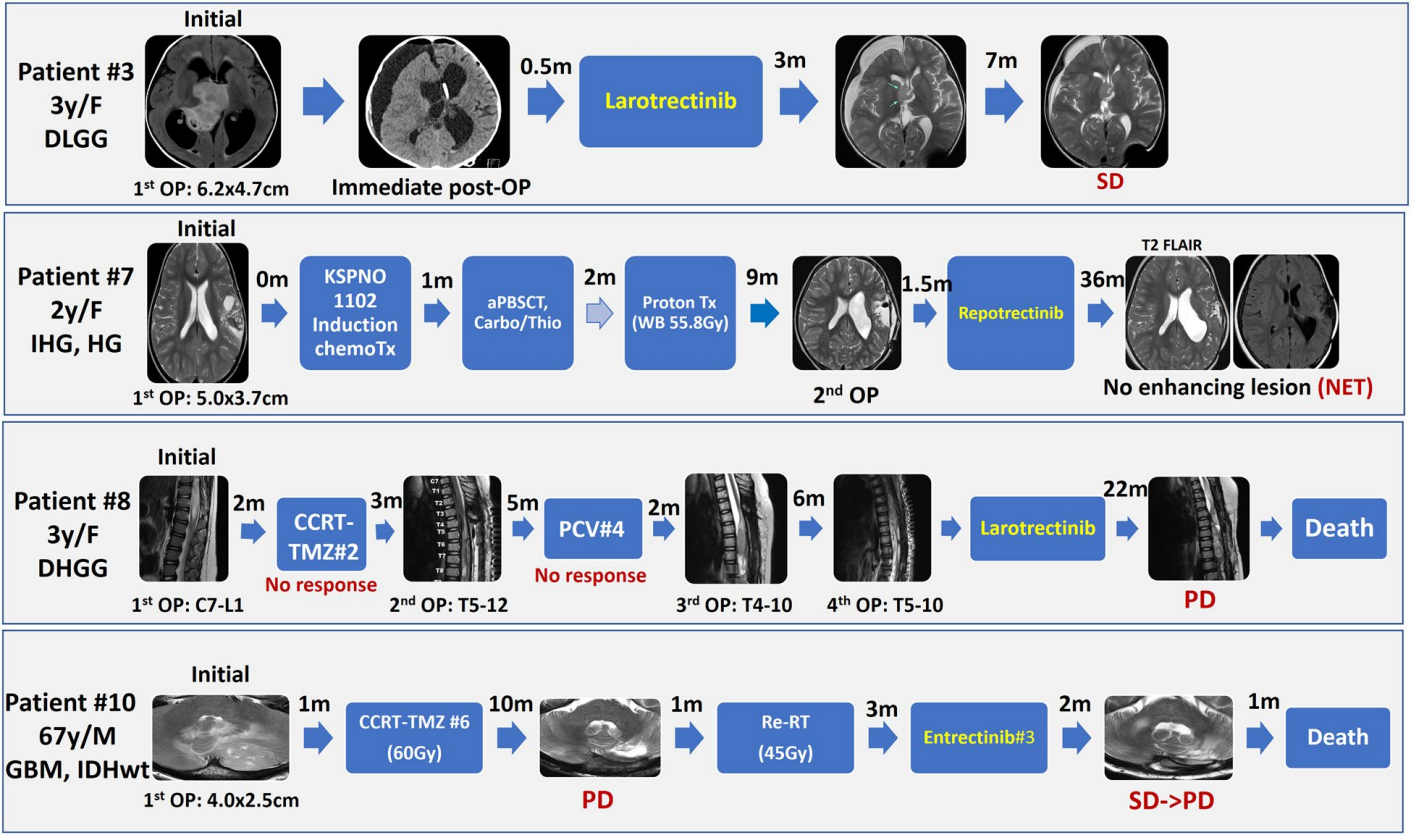
Summary of the radiological characteristics and treatment timelines for patients receiving TRK inhibitor therapy (IHG: Infant-type hemispheric glioma, DLGG: Diffuse low-grade glioma, DHGG: Diffuse high-grade glioma, GBM: Glioblastoma, IDH-Wild-type, OP: Operation, SD: Stable disease, PD: Progressive disease, NET: No evidence of tumour, aPBSCT: autologous peripheral blood stem cell transplantation; Carbo, carboplatin, Thio, thioflavin #3)

Four patients with LGG who underwent complete resection (patients #1, 2, 5, and 6) did not undergo adjuvant treatment. The patients had NET throughout their follow-up periods. However, one patient with residual tumour who was diagnosed with LGG (patient #3) and three patients with HGG (patients #5, #6, and #9) received TRK inhibitor therapy at different stages of disease management. The patients with NTRK-fused gliomas exhibited responses to TRK inhibitor therapy, though the durability of these responses varied among the patients (Fig. 5).

Patient #3, a 3-year-old female with *GKAP1::NTRK2-*fused LGG in the right thalamus and 3rd ventricle, underwent adjuvant larotrectinib treatment for residual tumour. During the 14-month follow-up period, MRI scans revealed stable disease. Larotrectinib treatment is ongoing and planned to be continued for an additional 10 months.

Patient #7, a 2-year-old female who was diagnosed with IHG with the *ZBTB43::NTRK2* fusion and hemizygous deletion of *CDKN2A/2B*, underwent induction chemotherapy, autologous peripheral blood stem cell transplantation (aPBSCT) with carboplatin and thiotepa, and proton therapy (55.8 Gy) following the initial surgical removal of her left frontotemporal tumour (Fig. 5). After the second surgery to remove the residual tumour, she was enrolled in an oral repotrectinib clinical trial. Throughout the 3-year follow-up period after repotrectinib treatment, this patient demonstrated NET. However, the final outcome of this patient is unknown, as the clinical trial has not yet been completed.

Patient #8, a 3-year-old girl who was previously healthy, presented at the clinic with sudden lower extremity weakness for the previous two weeks. Initial MRI of the spine revealed a tumour at the C7 to L1 level (Fig. 5). Following laminectomy and tumour removal, the patient was diagnosed with a spinal GBM, IDH-wt, by the 2016 WHO criteria. Within one month after the operation, the patient developed gait disturbance and constipation. Only four months after the initial operation and following two cycles of CCRT-TMZ, a second laminectomy and intramedullary removal of the residual tumour were conducted (Fig. 5). After pathological confirmation of the recurrence as a spinal GBM-like HGG, four cycles of procarbazine, lomustine, and vincristine (PCV) chemotherapy were administered. As the tumour recurred again during follow-up, a third operation was conducted. During this procedure, SNUH utilized NGS with a brain tumour-targeted gene panel, identifying the *TPM3::NTRK1* fusion, amplification of *MDM4/AKT3*, and one-copy loss of *PTEN/FGFR2*, leading to the diagnosis of the tumour as HGG, not otherwise specified (NOS), according to the WHO2021 criteria. Subsequent recurrences warranted a fourth operation, and after the fifth recurrence, a decision was made to enrol the patient in a clinical trial for larotrectinib. She was resistant to the offered chemotherapies and had stable disease with larotrectinib. However, the patient passed away due to disease progression 22 months after the initial trial of larotrectinib.

Patient #9, a 64-year-old male with *SPECC1L::NTRK2-*fused HGG with additional homozygous deletion of *CDKN2A*, underwent adjuvant CCRT-TMZ only. Despite ependymal enhancement, the condition of patient #9 remained relatively stable without gait disturbance during the 27-month follow-up.

Patient #10, a 67-year-old male with *FKBP15::NTRK2-*fused HGG of the cerebellum, had additional *PDGFRA/KIT* amplification, homozygous deletion of *CDKN2A/2B* and 1-copy loss of *PTEN/NF1*. He received entrectinib after completing CCRT-TMZ#6 and re-radiation therapy (45 Gy). The tumour showed transiently stable disease on entrectinib for 2 months, but the patient eventually experienced disease progression and death in three months after starting entrectinib (Table 3 and Fig. 5).

Patients #11 and #12 did not undergo additional treatment after surgery. Patient #11, a 72-year-old male with the *KANT::TRAK2* fusion and the associated histopathological characteristics, along with additional genetic abnormalities – homozygous deletion of *CDKN2A/2B*, mutation of *PTEN/TP53*, *TERTp* mutation (C228T), *PTEN* loss, trisomy 7, and losses of 10, 14q, 22q, indicative of HGG – was in compromised general health and died 4 months after surgery.

Patient #12, a 54-year-old male diagnosed with glioblastoma (GBM) with the *BCR::NTRK2* fusion, PTEN mutation, homozygous deletion of *CDKN2A/2B*, and *TERTp* mutation (C228T), refused adjuvant therapy and received palliative care, resulting in death 13 months post-surgery.

## Discussion

The development and evolution of first-generation TRK inhibitors and the subsequent development of second-generation TRK inhibitors represent significant milestones in precision oncology [29]. First-generation TRK inhibitors were initially developed following the discovery of NTRK family gene fusions across diverse cancers. Their development process included rigorous preclinical studies confirming their efficacy against NTRK fusion proteins [7]. Their efficacy and safety were evaluated in subsequent clinical trials, resulting in their regulatory approval [6, 15, 24, 29]. First-generation TRK inhibitors, such as larotrectinib and entrectinib, effectively block downstream signalling pathways, resulting in durable responses and favourable tolerability, as observed in clinical trials [15]. Both larotrectinib and entrectinib function as TRK inhibitors by targeting the ATP binding site of NTRK family genes. Additionally, entrectinib has a broader spectrum of activity than other first-generation TRK inhibitors, also targeting *ROS1* and *ALK* fusion genes through the same binding mechanism. Drugs acting through both mechanisms have shown high response rates in clinical trials; however, some patients have developed resistance to these drugs over time [29]. The development of second-generation TRK inhibitors, such as repotrectinib, aimed to overcome these resistance mechanisms and provide therapeutic options for patients who experience relapse or develop resistance to first-generation TRK inhibitors. Repotrectinib targets multiple kinases, including NTRK, ROS1, and ALK [24]. Second-generation NTRK inhibitors utilize an allosteric binding site other than the ATP binding site and thus interact with regions outside the ATP binding site to regulate TRK fusion activity. This method increases their selectivity for TRK fusion proteins, minimizing off-target effects and reducing the risk of adverse reactions [24]. Other second-generation TRK inhibitors, such as selitrectinib and taletrectinib, have shown increased efficacy in patients who have experienced progression on first-line therapies or who have developed resistance mutations. Clinical trials for these drugs are currently in progress, aiming to further explore their potential for overcoming resistance to targeted therapy [29].

The primary recognized adverse effects of TRK inhibitors are constitutional symptoms, including nausea, vomiting, diarrhoea, liver toxicity, peripheral oedema, rash, cardiac toxicity, and neurological effects such as dizziness, headache, or peripheral neuropathy [26, 27]. Despite these adverse effects, TRK inhibitors are generally well tolerated by patients.

Among the twelve patients diagnosed with CNS gliomas harbouring *NTRK* fusions, four received TRK inhibitors; three of these were paediatric patients, and one was an adult patient. The choice of TRK inhibitor for each patient was determined solely by the clinical trials in which they were enrolled. One adult patient (#10) received entrectinib after completing CCRT with six cycles of TMZ. The patient initially had stable disease for 2 months; however, no significant therapeutic benefit was observed. Disease progression occurred, and the patient eventually died 3 months after starting entrectinib treatment.

The question of whether initiating a TRK inhibitor as the first-line adjuvant chemotherapy is equivalent to or beneficial compared to the conventional CCRT-TMZ#6 (6 treatment cycles) Stupp protocol remains unanswered [19]. Particularly in paediatric patients, the use of a CCRT-TMZ#6 adjuvant regimen, which is the standard approach for managing HGG in adults, requires careful consideration. This approach has significant concerns due to its high toxicity, the potential for the development of secondary tumours in response to radiation therapy, and its adverse effects on development and growth [23]. Considering these concerns, paediatric patients with CNS tumours are ideal candidates for treatment with a TRK inhibitor as first-line adjuvant chemotherapy. The relatively mild toxicity profile associated with targeted therapies makes this approach an attractive option for paediatric patients. For instance, patient #3 (3 y/F) received larotrectinib for residual DLGG after surgery. During her 10-month evaluation period, she maintained stable disease, with a progression-free survival (PFS) time of at least 10 months.

Patient #7 (2 y/F), who was diagnosed with *ZBTB43::NTRK2-*fused HGG, underwent induction chemotherapy, aPBSCT with carboplatin and thiotepa, and proton therapy (55.8 Gy) after her first surgery. Unfortunately, due to tumour progression, a second surgical intervention was performed. Following the second gross total removal of the tumour, she started treatment with repotrectinib. Remarkably, after completing the repotrectinib regimen, there was no evidence of tumour recurrence for 3 years. This exceptional response in a HG IHG highlights the prominent potential of targeted TRK inhibitor therapy in such challenging cases. However, the exact outcome of this patient remains undetermined, as the clinical trial in which she is enrolled is ongoing. Relative to the other patients, this patient had only hemizygous deletion of CDKN2A/2B as an additional genetic alteration, which is thought to have contributed to her relatively good prognosis.

Importantly, 40% of nonbrainstem HGGs in children less than 3 years old have been reported to harbour an *NTRK* fusion [31]. This rate is significantly greater than the *NTRK* fusion rate of approximately 1% in CNS tumours. However, it is challenging to compare survival gains specific to IHG following treatment with TRK inhibitors, due to the rarity of this tumour type. However, regardless of tumour grade, the 5-year overall survival (OS) rate for this type of tumour is 42.9% [12]. Considering that the tumour in patient #7 was classified as CNS WHO grade 4, her disease-free survival time of 36 months following treatment with repotrectinib is especially remarkable.

The case of patient #8 (2 y/F), who had a *TPM3::NTRK1*-fused spinal HGG, highlights the potential efficacy of TRK inhibitor (larotrectinib) therapy in the management of recurrent and challenging tumours [21]. This tumour was diagnosed as GBM in 2017 by the revised WHO2016 classification, and the patient underwent CCRT-TMZ. This type of fusion, previously identified in soft tissue sarcoma and nonbrainstem paediatric HGGs [11, 28, 31], had never been documented in the spinal cord before this case. However, this patient had additional genetic abnormalities, such as MDM4/AKT3 amplification, and experienced multiple recurrences. After the fifth tumour recurrence, the patient was enrolled in a larotrectinib clinical trial, but she died due to tumour progression 22 months after starting larotrectinib.

A French nationwide study reported a median OS time of 13.1 months and a median PFS time of 8.0 months for primary spinal GBM among a generally older population (mean age: 37 years, standard deviation (SD): 16.5). Remarkably, patient #8 achieved an OS time of 22 months post-larotrectinib treatment, significantly exceeding the OS time observed in the French study [1].

The outcomes of these patients demonstrate the notable potential of TRK inhibitors in managing rare and complex cases of tumours such as primary spinal HGG, which accounts for approximately 0.1% of gliomas.

The tumour grade and additional molecular alterations play crucial roles in determining the potential value of *NTRK*-targeted therapy. Patients with DHGG with additional molecular features alongside *NTRK* fusion eventually experienced disease progression despite undergoing therapy. Conversely, patients with DLGG without additional genetic alteration who were treated with a TRK inhibitor had stable disease. This pattern indicates that while NTRK-targeted therapies can be effective, their success may vary significantly depending on the tumour grade and the presence of other molecular alterations.

The optimization of *NTRK* fusion screening using pan-TRK IHC demonstrated high sensitivity, as shown in the 2020 Belgian Ring trial [4] and 2024 CanTRK trial [14], both of which were conducted with the same clones used in our study. However, variability in staining intensity and staining patterns, influenced by different fusion partners, was noted; this variability posed a challenge, especially in CNS tumours, where normal brain tissue also shows pan-TRK IHC positivity [17]. Despite these challenges, our study of twelve tumours harbouring *NTRK1* or *NTRK2* fusions revealed strong correlations and consistent positive results across various fusion partners. Karakas et al. reported that the sensitivity and specificity of pan-TRK IHC for *NTRK* fusions were 100% and 88%, respectively [18]. Moreover, the pan-TRK IHC concordance tends to be lower for *NTRK3* fusions than for *NTRK1* and *NTRK2* fusions [11].

Except for a paediatric spinal HGG (GBM-like) and a DIG, which harboured *NTRK1* fusions, all other CNS tumours in this study exhibited *NTRK2* fusion, consistent with its status as the most common NTRK family member exhibiting fusion in CNS tumours [11]. Importantly, a distinct fusion partner was found in each case, highlighting the molecular heterogeneity within the studied CNS tumours. This observation emphasizes the need for individualized approaches for understanding and targeting specific *NTRK* fusions in the clinical setting, as well as the necessity of NGS for a final diagnosis.

We report *ZBTB43::NTRK2* as a new fusion that has not been previously reported in any tumour. This fusion was identified in the HG IHG of patient #8 (3 y/F) diagnosed according to the WHO2021 diagnostic criteria. The NTRK-fused cerebral gliomas in paediatric patients, with WHO grades ranging across the scale, exhibited no additional genetic alterations. This feature contrasts sharply with the findings in adult GBMs, and this observation highlights the existence of NTRK-fused DLGGs and teaches neuropathologists to be alert in identifying *NTRK* fusions. Moreover, this need for caution extends beyond HG IHGs to encompass a wide range of gliomas, irrespective of their grade or age at onset.

Torre et al*.* reported that NTRK-fused gliomas in paediatric patients are of various histologic types, such as DIG, pilocytic astrocytoma, ganglioglioma, and GBM. Our cases included a variety of histopathological classifications, such as IHG, MGNT of the lateral ventricle, astrocytoma with neuropil-like islands, spinal HGG, and GBM. Thus, the histopathology of NTRK-fused CNS tumours is characterized by heterogeneity.

Additionally, our NTRK-fused gliomas were identified across a wide age range of patients and in various locations, including the cerebrum, thalamus, intraventricular area, cerebellum, and spinal cord. Therefore, importantly, NTRK-fused glioma are not exclusively IHG, a paediatric-type DHGG.

## Conclusion

The diverse presentations of NTRK-fused CNS tumours complicate the process of diagnosis according to the WHO2021 criteria. However, the therapeutic potential of TRK inhibitors is evident in the positive outcomes obtained. Considering the favourable outcomes of patients undergoing TRK inhibitor therapy, routine screening of all CNS tumours is recommended, particularly in children. Bearing in mind its relatively low specificity, the incorporation of routine pan-TRK IHC testing has the potential to improve both diagnostic and therapeutic aspects of the management of CNS tumours.

### Supplementary Information

Supplementary material 1.

## Data Availability

The datasets used and/or analysed during the current study are available from the corresponding author upon reasonable request.

## References

[1] Amelot A, Terrier LM, Mathon B, Joubert C, Picart T, Jecko V, Bauchet L, Bernard F, Castel X, Chenin L (2023). Natural course and prognosis of primary spinal glioblastoma: a nationwide study. Neurology.

[2] Bourhis A, Caumont C, Quintin-Roue I, Magro E, Dissaux G, Remoue A, Le Noac'h P, Douet-Guilbert N, Seizeur R, Tyulyandina A (2022). Detection of NTRK fusions in glioblastoma: fluorescent in situ hybridisation is more useful than pan-TRK immunohistochemistry as a screening tool prior to RNA sequencing. Pathology.

[3] de Carvalho C, Correa D, Tesser-Gamba F, Dias Oliveira I, Saba da Silva N, Capellano AM, de Seixas Alves MT, Dastoli PA, Cavalheiro S, Caminada de Toledo SR (2022). Gliomas in children and adolescents: investigation of molecular alterations with a potential prognostic and therapeutic impact. J Cancer Res Clin Oncol.

[4] De Winne K, Sorber L, Lambin S, Siozopoulou V, Beniuga G, Dedeurwaerdere F, D'Haene N, Habran L, Libbrecht L, Van Huysse J (2021). Immunohistochemistry as a screening tool for NTRK gene fusions: results of a first Belgian ring trial. Virchows Arch.

[5] Doebele RC, Drilon A, Paz-Ares L, Siena S, Shaw AT, Farago AF, Blakely CM, Seto T, Cho BC, Tosi D (2020). Entrectinib in patients with advanced or metastatic NTRK fusion-positive solid tumours: integrated analysis of three phase 1–2 trials. Lancet Oncol.

[6] Downing NS, Aminawung JA, Shah ND, Krumholz HM, Ross JS (2014). Clinical trial evidence supporting FDA approval of novel therapeutic agents, 2005–2012. JAMA.

[7] Drilon A (2019). TRK inhibitors in TRK fusion-positive cancers. Ann Oncol.

[8] Drilon A, Laetsch TW, Kummar S, DuBois SG, Lassen UN, Demetri GD, Nathenson M, Doebele RC, Farago AF, Pappo AS (2018). Efficacy of larotrectinib in TRK fusion-positive cancers in adults and children. N Engl J Med.

[9] FDA US (2023) FDA approves repotrectinib for ROS1-positive non-small cell lung cancer https://www.fda.gov/drugs/resources-information-approved-drugs/fda-approves-repotrectinib-ros1-positive-non-small-cell-lung-cancer. Accessed January 28, 2024 2024

[10] Gambella A, Senetta R, Collemi G, Vallero SG, Monticelli M, Cofano F, Zeppa P, Garbossa D, Pellerino A, Ruda R (2020). NTRK fusions in central nervous system tumors: a rare, but worthy target. Int J Mol Sci.

[11] Gatalica Z, Xiu J, Swensen J, Vranic S (2019). Molecular characterization of cancers with NTRK gene fusions. Mod Pathol.

[12] Guerreiro Stucklin AS, Ryall S, Fukuoka K, Zapotocky M, Lassaletta A, Li C, Bridge T, Kim B, Arnoldo A, Kowalski PE (2019). Alterations in ALK/ROS1/NTRK/MET drive a group of infantile hemispheric gliomas. Nat Commun.

[13] Hong L, Shi ZF, Li KK, Wang WW, Yang RR, Kwan JS, Chen H, Li FC, Liu XZ, Chan DT (2022). Molecular landscape of pediatric type IDH wildtype, H3 wildtype hemispheric glioblastomas. Lab Invest.

[14] Hyrcza MD, Martins-Filho SN, Spatz A, Wang HJ, Purgina BM, Desmeules P, Park PC, Bigras G, Jung S, Cutz JC (2024). Canadian multicentric Pan-TRK (CANTRK) immunohistochemistry harmonization study. Mod Pathol.

[15] Jiang Q, Li M, Li H, Chen L (2022). Entrectinib, a new multi-target inhibitor for cancer therapy. Biomed Pharmacother.

[16] Jiang T, Wang G, Liu Y, Feng L, Wang M, Liu J, Chen Y, Ouyang L (2021). Development of small-molecule tropomyosin receptor kinase (TRK) inhibitors for NTRK fusion cancers. Acta Pharm Sin B.

[17] Kang J, Park JW, Won JK, Bae JM, Koh J, Yim J, Yun H, Kim SK, Choi JY, Kang HJ (2020). Clinicopathological findings of pediatric NTRK fusion mesenchymal tumors. Diagn Pathol.

[18] Karakas C, Giampoli EJ, Love T, Hicks DG, Velez MJ (2024). Validation and interpretation of Pan-TRK immunohistochemistry: a practical approach and challenges with interpretation. Diagn Pathol.

[19] Kim BS, Seol HJ, Nam DH, Park CK, Kim IH, Kim TM, Kim JH, Cho YH, Yoon SM, Chang JH (2017). Concurrent chemoradiotherapy with temozolomide followed by adjuvant temozolomide for newly diagnosed glioblastoma patients: a retrospective multicenter observation study in Korea. Cancer Res Treat.

[20] Kim H, Lim KY, Park JW, Kang J, Won JK, Lee K, Shim Y, Park CK, Kim SK, Choi SH (2022). Sporadic and Lynch syndrome-associated mismatch repair-deficient brain tumors. Lab Invest.

[21] Langston RG, Wardell CP, Palmer A, Scott H, Gokden M, Pait TG, Rodriguez A (2021). Primary glioblastoma of the cauda equina with molecular and histopathological characterization: case report. Neurooncol Adv.

[22] Lanman T, Hayden Gephart M, Bui N, Toland A, Nagpal S (2021). Isolated leptomeningeal progression in a patient with NTRK Fusion+ uterine sarcoma: a case report. Case Rep Oncol.

[23] Lee JH, Eom KY, Phi JH, Park CK, Kim SK, Cho BK, Kim TM, Heo DS, Hong KT, Choi JY (2021). Long-term outcomes and sequelae analysis of intracranial germinoma: need to reduce the extended-field radiotherapy volume and dose to minimize late sequelae. Cancer Res Treat.

[24] Liu F, Wei Y, Zhang H, Jiang J, Zhang P, Chu Q (2022). NTRK fusion in non-small cell lung cancer: diagnosis, therapy, and TRK inhibitor resistance. Front Oncol.

[25] Mohamed F, Kurdi M, Baeesa S, Sabbagh AJ, Hakamy S, Maghrabi Y, Alshedokhi M, Dallol A, Halawa TF, Najjar AA (2022). The diagnostic value of Pan-Trk expression to detect neurotrophic tyrosine receptor kinase (NTRK) gene fusion in CNS tumours: a study using next-generation sequencing platform. Pathol Oncol Res.

[26] Qin H, Patel MR (2022). The challenge and opportunity of NTRK inhibitors in non-small cell lung cancer. Int J Mol Sci.

[27] Shyam Sunder S, Sharma UC, Pokharel S (2023). Adverse effects of tyrosine kinase inhibitors in cancer therapy: pathophysiology, mechanisms and clinical management. Signal Transduct Target Ther.

[28] Solomon JP, Linkov I, Rosado A, Mullaney K, Rosen EY, Frosina D, Jungbluth AA, Zehir A, Benayed R, Drilon A (2020). NTRK fusion detection across multiple assays and 33,997 cases: diagnostic implications and pitfalls. Mod Pathol.

[29] Theik NWY, Muminovic M, Alvarez-Pinzon AM, Shoreibah A, Hussein AM, Raez LE (2024). NTRK therapy among different types of cancers. Int J Mol Sci Rev Fut Perspect.

[30] Woo HY, Na K, Yoo J, Chang JH, Park YN, Shim HS, Kim SH (2020). Glioblastomas harboring gene fusions detected by next-generation sequencing. Brain Tumor Pathol.

[31] Wu G, Diaz AK, Paugh BS, Rankin SL, Ju B, Li Y, Zhu X, Qu C, Chen X, Zhang J (2014). The genomic landscape of diffuse intrinsic pontine glioma and pediatric non-brainstem high-grade glioma. Nat Genet.

[32] Xiang S, Lu X (2024). Selective type II TRK inhibitors overcome xDFG mutation mediated acquired resistance to the second-generation inhibitors selitrectinib and repotrectinib. Acta Pharm Sin B.

[33] Yang Y, Li S, Wang Y, Zhao Y, Li Q (2022). Protein tyrosine kinase inhibitor resistance in malignant tumors: molecular mechanisms and future perspective. Signal Transduct Target Ther.

