# Supplementary material for: NTRK-fused central nervous system tumours: clinicopathological and genetic insights and response to TRK inhibitors
Source: Acta Neuropathol Commun. 2024 Jul 16;12:118. doi: 10.1186/s40478-024-01798-9 (PMC11251294; doi:10.1186/s40478-024-01798-9)
Supplement: Supplementary file 1 — Supplementary material 1. [file 40478_2024_1798_MOESM1_ESM.docx]

Supplementary Table 1. The primary antibodies used in this study.

| Antibody | Dilution | Antigen retrieval | Clone | Source |
| --- | --- | --- | --- | --- |
| ATRX | 1: 200 | Ventana CC1 100^o^C | Polyclonal | ATLAS ANTIBODIES AB, Bromma,Sweden |
| BRAF | 1:200 | Ventana CC1 100^o^C | VE1 (monoclonal) | Spring Bioscience, CA, US |
| GFAP | 1: 200 | Ventana CC1 100^o^C | 6F2 (monoclonal) | DAKO, Glostrup, Denmark |
| IDH-1 | 1: 100 | Ventaan CC1 100^o^C | H09 (monoclonal) | Dainova, Hamburg, Germany |
| K27M | 1: 1000 | Ventana CC1 100^o^C | HH3 (monoclonal) | Milipore, Temecula, US |
| Ki67 | 1: 100 | Ventana CC1 100^o^C | MIB-1 (monoclonal) | DAKO, Glostrup, Denmark |
| TRK | RTU | Ventana CC1 100^o^C | EPR17341 | Ventana, Export, US |
| TRK | 1: 50 | Ventana CC1 100^o^C | A7H6R | Cell signaling, Massachusetts, US |
| Olig2 | 1: 500 | Ventana CC1 100^o^C | 211F1.1 | Cell Marque, Rocklin, US |
| P53 | 1: 1000 | Ventana CC1 100^o^C | DO7 (monoclonal) | DAKO, Glostrup, Denmark |
| pHH3 | 1: 100 | Ventana CC1 100^o^C | Polyclonal | Cell Marque, Rocklin, US |
| Synaptophysin | 1: 200 | Bond H2O ER2 200^o^C | 27G12 (monoclonal) | NOVO, Newcastle, UK |
| NeuN | 1: 500 | Ventana CC1 100^o^C | A60 (monoclonal) | Millipore, Temecula, US |

GFAP, glial fibrillary acidic protein; ATRX, Alpha Thalassemia associated mental retardation X; K27M, Histon lysin27methionine; IDH-1, isocitrate dehydrogenase 1; pHH3, phosphorylated Histone H3; RTU, ready to use

Supplementary Table 2. Detailed histopathology, provisional grade, immunohistochemical findings, and integrated diagnosis of our NTRK-fused glioma.

| No. | Sex | Age (year) | Gene fusion | Grade | Histopathological findings | GFAP | Olig2 | Synap | panTrk | Mitoses/10HPFs | MVP | Necrosis | Ki-67 inex | Integrated diagnosis |
| --- | --- | --- | --- | --- | --- | --- | --- | --- | --- | --- | --- | --- | --- | --- |
| 1 | F | 14 | *HOOK3::NTRK2* | 1 | DLGG | P | n | P | P | 0 | no | no | 0.6% | DLGG, NTRK-altered, NEC |
| 2 | F | 15 | *KIF5A::NTRK2* | 1 | Myxoid GNT-like | P | p | p | p | 1 | no | no | 5.7% | DLGG, NTRK-altered, NEC |
| 3 | F | 3 | *GKAP1::NTRK2* | 1 | DLGG | P | n | s | p | 1 | no | no | 4.4% | DLGG, NTRK-altered, NEC |
| 4 | F | 1 | *TPR::NTRK1* | 1 | DIG | P | P | fP | p | 2 | no | no | 3.0% | DLGG, NTRK-altered, NEC |
| 5 | F | 27 | *LHFPL2::NTRK2* | LG | Astrocytic tumor with neuropil-like islands | fP | P | fP | p | 4 | no | no | 1.2% | DLGG, NTRK-altered, NEC |
| 6 | F | 31 | *SLMAP-NTRK2* | 1 | DLGG | P | fP | n | p | 0 | no | no | 2.4% | DLGG, NTRK-altered, NEC |
| 7 | F | 2 | *ZBTB43::NTRK2* | 4 | GBM-like | P | P | ND | P | 18 | p | p | 27.8% | IHG, NTRK-altered |
| 8 | F | 2 | *TPM3::NTRK1* | 4 | GBM-like | P | P | n | p | 18 | p | p | 60.95% | DHGG, NTRK-altered, NEC |
| 9 | M | 64 | *SPECC1L::NTRK2* | 4 | GBM-like | P | P | n | p | 45 | p | p | 35.8% | GBM, IDH-wt, NOS |
| 10 | M | 67 | *FKBP15::NTRK2* | 4 | GBM-like | P | P | n | p | 59 | p | p | 33.0% | GBM, IDH-wt, NOS |
| 11 | M | 72 | *KANK1::NTRK2* | 4 | GBM-like | P | P | n | p | 82 | p | p | 90.0% | GBM, IDH-wt, NOS |
| 12 | M | 54 | *BCR::NTRK2* | 4 | GBM-like | P | P | n | p | 90 | p | p | 88.4% | GBM, IDH-wt, NOS |

MVP, microvascular proliferation; DLGG, Diffuse low grade glioma; MGNT, myxoid glioneuronal tumor; DIG, desmoplastic infantile ganglioglioma; IHG, infant-type hemispheric glioma; GBM, glioblastoma, IDH-wildtype; p, positive, fp, focal positive; n, negative, ND, not done, NEC, not elsewhere classified; NOS, not otherwise specified
